# Establishing the need and identifying goals for a curriculum in medical business ethics: a survey of students and residents at two medical centers in Missouri

**DOI:** 10.1186/1756-0500-7-708

**Published:** 2014-10-09

**Authors:** Elena M Kraus, Erin Bakanas, Kamal Gursahani, James M DuBois

**Affiliations:** Bander Center for Medical Business Ethics, Saint Louis University, Salus Center 3545 Lafayette Ave, Saint Louis, MO 63104-1314 USA; Department of Internal Medicine Division of General Internal Medicine, 12 South FDT, Saint Louis University School of Medicine, 1402 South Grand Blvd, Saint Louis, MO 63104 USA; Department of Surgery, Division of Emergency Medicine, Saint Louis University School of Medicine, 3635 Vista Avenue at Grand Blvd, Saint Louis, MO 63110 USA; Division of General Medical Sciences, Washington University School of Medicine, 660 South Euclid Ave, Box 8005, Saint Louis, MO 63110 USA

**Keywords:** Medical business ethics, Professional ethics, Clinical ethics, Medical ethics, Medical education, Graduate medical education, Business in medicine, Healthcare industry

## Abstract

**Background:**

In recent years, issues in medical business ethics (MBE), such as conflicts of interest (COI), Medicare fraud and abuse, and the structure and functioning of reimbursement systems, have received significant attention from the media and professional associations in the United States. As a result of highly publicized instances of financial interests altering physician decision-making, major professional organizations and government bodies have produced reports and guidelines to encourage self-regulation and impose rules to limit physician relationships with for-profit entities. Nevertheless, no published curricula exist in the area of MBE. This study aimed to establish a baseline level of knowledge and the educational goals medical students and residents prioritize in the area of MBE.

**Methods:**

732 medical students and 380 residents at two academic medical centers in the state of Missouri, USA, completed a brief survey indicating their awareness of major MBE guidance documents, knowledge of key MBE research, beliefs about the goals of an education in MBE, and the areas of MBE they were most interested in learning more about.

**Results:**

Medical students and residents had little awareness of recent and major reports on MBE topics, and had minimal knowledge of basic MBE facts. Residents scored statistically better than medical students in both of these areas. Medical students and residents were in close agreement regarding the goals of an MBE curriculum. Both groups showed significant interest in learning more about MBE topics with an emphasis on background topics such as “the business aspects of medicine” and “health care delivery systems”.

**Conclusions:**

The content of major reports by professional associations and expert bodies has not trickled down to medical students and residents, yet both groups are interested in learning more about MBE topics. Our survey suggests potentially beneficial ways to frame and embed MBE topics into the larger framework of medical education.

## Background

In the market context of medicine in the United States, issues in medical business ethics (MBE), such as conflicts of interest (COI), Medicare fraud and abuse, and the structure and functioning of reimbursement systems, affect the integrity of medical practice and research. Substantial data indicate that self-serving bias subconsciously influences the way physicians weigh information and make decisions when financial incentives exist [1]. Financial relationships between physicians and medical industry are ubiquitous [2] and have measurable effects on physician behavior [3, 4]. The business dimensions of medicine add complexity to physicians’ daily decisions about patient care [5, 6], and cases of physicians who prioritize profit over the safety and health of patients are regularly made public [7–10]. These cases undermine public trust, leading to questions about the validity of research results and clinical decisions.

The Institute of Medicine (IOM) [11], the Association of American Medical Colleges [12, 13], the American Medical Student Association [14], the Office of the Inspector General (OIG) [15], and the U.S. Congress [16], share these concerns as indicated by reports and guidelines on such topics published over recent years. Most encourage self-regulation by the profession while imposing rules that can be frustrating for clinicians and scientists [17].

Attitudes toward industry are formed early in medical training [18], suggesting the need for early MBE education. The Accreditation Council for Graduate Medical Education (ACGME) lists both professionalism (which includes ethics) and systems-based practice (which includes awareness of the larger context and system of health care and its resources) as two of their six core competencies of patient care [19]. Medical students and residents generally support initiatives in ethics and professionalism [20]. Nevertheless, many important ethical issues, such as Medicaid and Medicare fraud and abuse, are not uniformly presented to training physicians in the United States [21].

Furthermore, although there are no data to indicate graduating residents’ views, the majority of graduating medical students believe current instruction and the exposure they have in medical school to health policy and economics has poorly prepared them for entrance into the US healthcare system [22]. Knowledge of health policy and economics is only the beginning of understanding the myriad ethical issues intricately entwined in the financial relationships that are involved in medical practice and applying this understanding to professional practice. Current ethics education is lacking in both this foundational knowledge as well as its related ethical conundrums [23].

The AMSA PharmFree Scorecard Methodology rates academic medical centers on the quality of their policies and practices regarding conflicts of interest, including the provision of an adequate curriculum for medical students on conflicts of interest. They specifically examine whether “Students are trained to understand institutional conflict-of-interest policies and recognize how industry promotion can influence clinical judgment [24]”. While this concentrates attention on just one aspect of MBE, it reflects a growing awareness of the importance of MBE for medical ethics and professionalism over the past decade: In 2002, fewer than 1 in 4 academic medical center’s ethics curriculum dedicated attention to COIs (DuBois and Burkemper 2002).

The Bander Center for Medical Business Ethics at Saint Louis University (Bander Center), with whom the four authors are affiliated, has established the goal of publishing a freely available, online case-based curriculum in this area. This paper reports on a preliminary step in that process: Surveying medical students and residents to establish needs, interests, and priorities in the area of MBE.

## Methods

### Study population

In January and February 2013, a brief, confidential survey was emailed to medical students and residents at Saint Louis University (SLU) and Washington University (WU) in Saint Louis, Missouri. Among those invited, 720 were medical students at SLU, 576 were medical students at WU (n = 1296), 617 were residents at SLU, and 562 were residents at WU (n = 1179) for a total of 2475. These medical schools incorporate both scheduled and elective courses in healthcare ethics and health policy as part of the required medical doctorate curricula; formal ethics instruction at both institutions occurs primarily during the preclinical years (MS1 and MS2). Residents at these institutions have less formalized training in ethics that may include lectures and ethics continuing medical education (CME). The 2013 AMSA Scorecard on “Conflicts of Interest Policies in Academic Medical Centers” graded the policies at WU with an “A” and policies at SLU with a “B”.

### Questionnaire and distribution

A nine-item questionnaire was distributed using Qualtrics, a web-based survey software system. Invitations were sent over three weeks. Following the initial invitation, reminder emails were sent after the end of week one and week two to individuals who had not yet replied. As an incentive to complete the survey, we offered participants at each institution the chance to win an iPad Mini or one of two $50 gift cards; a total of six prizes were awarded using a lottery method.

Participants were asked to select their affiliated academic medical center and their level of medical training. They were asked how familiar they were with four major guidance documents in the area of MBE: The Institute of Medicine (IOM) report entitled “Conflict of Interest in Medical Research, Education, and Practice” [11]; The Office of Inspector General (OIG) self-study booklet entitled “A Roadmap for New Physicians: Avoiding Medicare and Medicaid Fraud and Abuse” [15]; The Pharmaceutical Research and Manufacturers of America (PhRMA) report entitled “Code on Interactions with Healthcare Professionals” [25]; and The Association of American Medical Colleges (AAMC) report entitled “In the Interest of Patients: Recommendations for Physician Financial Relationships and Clinical Decision Making” [12]. They could choose one of three answers: “not at all”, “heard of it”, or “familiar with its content”. We believed that familiarity with some of the reports that drove policy development within academic medical centers would reflect awareness of developments in the area of conflicts of interest and medical business ethics. This seemed appropriate given that literature on medical business ethics is still in its infancy, and the Institute of Medicine Report has been cited widely by the AAMC and has been used by the authors in educational sessions with medical students and residents.

The next four multiple-choice questions tested participants’ knowledge of key research on MBE: the prevalence of physician relationships with medical industry [2], the nature of self-serving bias [1], how much pharmaceutical companies spend on marketing free drug samples [26], and the Bayh-Dole Act of 1980, which allows government-funded research discoveries to be patented by research institutions and royalty income to be shared with investigators [27].

The final two questions captured medical students’ and residents’ beliefs about the goals of an education in MBE, and the areas of MBE they were most interested in learning more about.

This study was approved by the Institutional Review Board (IRB) at Saint Louis University.

### Analysis

Survey data were analyzed using the Statistical Package for the Social Sciences (SPSS). Statistical analysis focused on descriptive statistics and the comparison of medical students (MS) and post-graduate residents (PG) using Chi square.

## Results and discussion

The survey response rate was 45% (n = 1112). Respondents were evenly split between institutions: 553 from SLU (50%) and 559 from WU (50%). Respondents included 732 medical students (65%)—MS1 (16%), MS2 (16%), MS3 (12%), MS4 (13%), MD/PhD (8%)—and 380 residents (34%)—PGY1 (6%), PGY2 (7%), PGY3 (6%), PGY4 (5%), and PGY5 (4%), and PGY > 5 (6%).

Tables 1, 2, 3 and 4 present participant responses to the four areas of questioning, which are discussed below.

**Table 1:** **MBE awareness questions**

Item: How familiar are you with the following guidance documents?	MS %	PG %	X ^2^
Institute of Medicine’s Report entitled “Conflicts of Interest in Medical Research, Education, and Practice”			94.9**
Not at all	62.8%	33.4%	
Heard of it	31.3%	49.7%	
Familiar with content	5.9%	16.8%	
Office of the Inspector General’s self-study booklet entitled “A Roadmap for New Physicians: Avoiding Medicare and Medicaid Fraud and Abuse”			111.3**
Not at all	88.9%	63.2%	
Heard of it	10.8%	32.1%	
Familiar with content	.3%	4.7%	
The Pharmaceutial Research and Manufacturers of America (PhRMA) report entitled, “Code on Interactions with Healthcare Professionals”			74.9**
Not at all	85.8%	63.2%	
Heard of it	12.0%	30.8%	
Familiar with content	2.2%	6.1%	
The Association of American Medical Colleges (AAMC) report entitled “In the Interest of Patients: Recommendations for Physician Financial Relationships and Clinical Decision Making”			43.8**
Not at all	80.1%	64.5%	
Heard of it	18.9%	29.2%	
Familiar with content	1.1%	6.3%	

** = p < .01 MS n = 732, PG n = 380.

**Table 2:** **MBE knowledge questions**

Item stem with correct response in boldface	MS %	PG %	X ^2^
Estimate the percentage of academic physicians that have a financial relationship with industry? …			
5 – 15%	7.5%	11.8%	
15 – 25%	34.4%	41.6%	
25 – 50%	32.2%	28.9%	
**> 50%**	**14.1%**	**10.8%**	17.6**
I don’t know	7.7%	6.8%	
As noted in the AAMC and IOM documents on conflicts of interest in medicine, social science data suggests that			
**“Self-serving bias is difficult to manage through disclosure alone”.**	**49.2%**	**58.9%**	26.2**
“Disclosure of conflicts of interest is sufficient to protect patients’ interests”.	5.9%	10.5%	
“Most physicians will not disclose their conflicts of interest even when required”.	6.7%	6.1%	
“Physicians with antisocial personalities harm patients when they have conflicts of interest”.	0.7%	0.5%	
I don’t know	37.6%	23.9%	
What percentage of the marketing budget of pharmaceutical companies is spent on free drug samples? …			
10%	12.3%	15.8%	
25%	26.9%	32.9%	
**50%**	**19.7%**	**24.7%**	23.3**
75%	5.9%	3.2%	
I don’t know	35.2%	23.4%	
The Bayh-Dole Act of 1980 allows academic medical centers that receive government research funding to:			
“Collaborate with private industry in medical research”.	10.4%	18.7%	
“Retain the copyright on all scientific publications produced by faculty”.	4.1%	6.1%	
**“Patent discoveries made with government research funding”.**	**10.7%**	**14.5%**	27.2**
“Bill for faculty salaries at a rate equal to the highest government employee salary”.	0.3%	0.8%	
I don’t know	74.6%	60.0%	

** = p < .01 MS n = 732, PG n = 380.

**Table 3:** **Goals of learning about MBE**

Item: An education in medical business ethics is important insofar as it helps physicians to meet the following goals:	MS mean (SD)	PG mean (SD)	t
To avoid legal problems (e.g., avoid Medicare fraud).	1.83 (.75)	1.87 (.74)	-.81
To prioritize patients’ best interests over the financial interests of others (such as 3^rd^ party payers, industry, or physician practices).	1.71 (.79)	1.82 (.83)	-2.1*
To foster the common good by being good stewards of limited healthcare dollars.	1.93 (.81)	1.97 (.86)	-.90
To achieve their own financial goals as physicians without violating the law or rules for professionalism.	2.10 (.84)	2.12 (.83)	-.33

Responses used a 5-point Likert-type scale from 1 (strongly agree) to 5 (strongly disagree).

* = p < .05 MS n = 732, PG n = 380.

**Table 4:** **MBE areas of interest to participants**

Item: What areas of medical business ethics are you most interested in learning more about? ***(Check as many as apply)***	MS %	PG %	X ^2^
Physician-industry interactions in clinical practice (e.g., interactions with drug and device reps)	61.2%	41.3%	39.9**
Regulation of physician business practices (e.g. fraud laws)	39.3%	43.7%	2.0
Whistleblowing (e.g. when and how to report medical fraud)	31.0%	19.2%	17.7**
The business aspects of medicine (e.g., billing, coding, and reimbursement systems)	61.5%	69.5%	7.0**
Health care delivery systems (e.g., understanding managed care, employer-based insurance, single payer systems, etc.)	63.7%	54.5%	8.8**
Health care access, rationing, resource allocation	59.3%	42.6%	27.9**
Physician organizations and political activism	26.0%	20.5%	4.0*
Physicians as employees	38.8%	32.1%	4.8*
Physicians as owners of medical practices	41.4%	37.6%	1.5
Professionalism and self-regulation	24.7%	23.9%	.08
Conflicts of interest in medical research	33.7%	25.3%	8.4**
Physicians as owners of intellectual property and data	34.4%	34.7%	.01

* = p < .05 ** = p < .01 MS n = 732, PG n = 380.

### Awareness of major MBE reports

Overall, participants had little awareness of recent, major reports addressing MBE topics (see Table 1). The vast majority of medical students had never heard of any of the reports, and almost no students had familiarity with them. One third to half of residents had at least heard of some of the four reports; overall residents had significantly more awareness and familiarity with these reports than medical students.

### Knowledge of MBE

As indicated in Table 2, medical student and resident knowledge of basic MBE facts was minimal. Similar to the first set of questions on general awareness of major reports on MBE, residents knew significantly more than medical students (see Table 2).

The highest percentage of correct answers came in response to the second question: “As noted in the AAMC and IOM documents on conflicts of interest in medicine, social science data suggests that … ‘self-serving bias is difficult to manage through disclosure alone.’” Although the answer to this question may be more intuitive when compared to the other questions in this section testing knowledge, the fact that residents and medical students are aware of self-serving bias and its need to be managed more effectively is interesting, given that this thesis has been generally difficult for practicing physicians to accept [1]. In fact, physicians have often expressed that they are above these biases, and that they are able to make objective decisions despite relationships with industry that may pose a personal financial incentive [28, 29].

Of note, only 5 participants answered all 4 knowledge questions correctly. That is .4% of all participants, which is precisely the percent expected with random guessing (.25 × .25 × .25 × .25 = .004 or .4%).

### Goals of learning MBE topics

In contrast to the basic MBE awareness and knowledge questions, medical students and residents were in close agreement on what they believed the goals of an MBE curriculum should be (see Table 3). The most strongly endorsed goal for both groups was for physicians “to prioritize patients’ best interests over the financial interests of others (such as third party payers, industry, or physician practices)” (m = 1.73 on a 5 point scale with 1 being strongly agree). This prioritization suggests their interest in these topics is not merely in order to obey laws and rules, but to understand how to uphold a true level of professionalism and put their patients’ best interests first. Recent research of medical student exposure to and attitudes about pharmaceutical companies reinforces the uncertainty they feel about how to manage these conflicts [30], and that their attitudes can be shaped by the substantial contact they have with pharmaceutical marketing even during undergraduate medical education [18]. Their strong agreement with all goals presented indicates they view this knowledge as important to their medical education and that they are sensitive to the tension it places on their decision-making. These findings reinforce the recommendations of the 2008 AAMC report on “Industry Support of Medical Education”, which recommended that

Educational programs should be developed to raise the awareness among students, trainees, and faculty of challenges to professionalism presented by certain interactions with industry and to provide opportunities that help them build critical evaluation skills that reinforce high individual standards, norms, and behaviors. P. 11.

### MBE topics of interest

There was a significant amount of interest by both groups in learning more about numerous MBE topics (see Table 4). The topics with the most interest from both groups were “the business aspects of medicine (e.g., billing, coding, and reimbursement systems)”, and “health care delivery systems (e.g., understanding managed care, employer-based insurance, single payer systems, etc.)”. Students and residents were slightly more interested in learning the basics of the business of medicine than the related ethical issues. We included these “basics of business” topics in our list because a separate Delphi survey we conducted with leaders throughout the US indicated that business ethics instruction should include a foundation in the business aspects of medicine [31].

Both groups were least interested in learning about “physician organizations and political activism”, “professionalism and self-regulation”, and “whistleblowing”. These results indicate that some of the most compelling aspects of these issues are encountered as the individual physician interacts with the larger medical system; our participants were less concerned with the interaction of the profession as a whole with the healthcare delivery system.

Although many topics received high interest from both groups, there were statistically significant differences between medical students and residents on seven of twelve topics. In general, medical students indicated more interest in the listed topics than residents, perhaps due to differences in level of responsibility and specific duties at the clinical level, experience, knowledge, and exposure to these issues.

We additionally provided participants with a textbox to write in topics of interest. Six medical students wrote in topics, some of which were new, and some of which specified topics in our list: Personal charity and free service; getting government out of healthcare regulation; transparency in medical billing; unethical retention of research data by pharmaceutical companies; physician entrepreneurship in an area outside of practicing medicine directly; the need to spend more time with patients even though fee for service medicine discourages this. No residents chose to write in a response.

## Conclusions

Our survey is limited by a response rate of 45%. Although a higher response rate would have been desirable, it seems unlikely that nonresponders would have been more familiar with MBE—one would expect interest in survey participation would increase if it were a topic familiar to the individual. Our survey is also limited by the number of items. A greater number of items would have more reliably assessed participants’ knowledge of MBE topics. However, a longer questionnaire would likely have reduced response rates. Moreover, the answers to the knowledge items selected were representative of facts frequently mentioned in the major reports on MBE that provided a focus for our project. We would also emphasize that our project focused only on education in medical business ethics. As the AMSA scorecard criteria rightly observe, this is just one component to navigating successfully business ethics in medicine, a component that must be supplemented by appropriate policies, mentoring, and practices.

### Identifying needs and interests

When groups such as the AAMC or IOM produce reports on matters such as conflicts of interest, it is often with the purpose of educating the profession and providing guidance. Our survey results indicate that these reports are not reaching young professionals who are supposed to benefit from their content. Residents were more knowledgeable than medical students and more aware of major reports on MBE, indicating that residency provides some exposure to such information, but overall the impact of resident experience alone is modest. These findings help to justify an increased focus on MBE topics within medical training programs.

### Framing a curriculum in MBE

The findings of this survey not only demonstrate a need for MBE education, but also provide guidance on how best to frame such a curriculum to meet the needs and interests of medical students and residents.

Curricula should frame MBE topics in terms of their impact on patient care. Doing so is not only consistent with guidance from the AAMC [13], but also reflects the priorities of medical students and residents.

We also recommend that the MBE curriculum be embedded in instruction on health care systems rather than in general medical ethics courses. This reflects the greater interest participants expressed in learning the basics of the business of medicine (health care delivery and reimbursement systems) than the related, more overtly ethical issues. But it also makes excellent sense from the perspective of ethics pedagogy. Combining the business aspects of medical practice with related ethical issues situates ethical theory in real-world dilemmas and reinforces the idea that ethics is not separate from medical professionalism, but is an essential dimension of it [17, 32].

## Authors’ information

All authors are affiliated with the Bander Center for Medical Business Ethics at Saint Louis University whose mission is to promote ethical business practices in medical care and research through the development of training and investigation opportunities for medical students, residents and physicians in practice. Information on the Center is available at: http://www.slu.edu/bander-center-home.

## Abbreviations

ACGME: Accreditation Council for Graduate Medical Education
COI: Conflict of interest
IRB: Internal Review Board
MBE: Medical business ethics
SLU: Saint Louis University
WU: Washington University.
